# Impact of Lowland Rainforest Transformation on Diversity and Composition of Soil Prokaryotic Communities in Sumatra (Indonesia)

**DOI:** 10.3389/fmicb.2015.01339

**Published:** 2015-12-08

**Authors:** Dominik Schneider, Martin Engelhaupt, Kara Allen, Syahrul Kurniawan, Valentyna Krashevska, Melanie Heinemann, Heiko Nacke, Marini Wijayanti, Anja Meryandini, Marife D. Corre, Stefan Scheu, Rolf Daniel

**Affiliations:** ^1^Genomic and Applied Microbiology and Göttingen Genomics Laboratory, Institute of Microbiology and Genetics, Georg-August-University Göttingen, Germany; ^2^Soil Science of Tropical and Subtropical Ecosystems, Buesgen Institute, Georg-August University Göttingen, Germany; ^3^Department of Soil Science, Faculty of Agriculture, University of Brawijaya Malang, Indonesia; ^4^Animal Ecology, J.F. Blumenbach Institute of Zoology and Anthropology, Georg-August University Göttingen, Germany; ^5^Department of Biology, Faculty of Mathematics and Natural Sciences IPB, Bogor Agricultural University Bogor, Indonesia

**Keywords:** rainforest conversion, soil microbial community composition, soil prokaryotic diversity, 16S rRNA gene, soil bacteria, soil archaea, oil palm, Sumatra

## Abstract

Prokaryotes are the most abundant and diverse group of microorganisms in soil and mediate virtually all biogeochemical cycles in terrestrial ecosystems. Thereby, they influence aboveground plant productivity and diversity. In this study, the impact of rainforest transformation to intensively managed cash crop systems on soil prokaryotic communities was investigated. The studied managed land use systems comprised rubber agroforests (jungle rubber), rubber plantations and oil palm plantations within two Indonesian landscapes Bukit Duabelas and Harapan. Soil prokaryotic community composition and diversity were assessed by pyrotag sequencing of bacterial and archaeal 16S rRNA genes. The curated dataset contained 16,413 bacterial and 1679 archaeal operational taxonomic units at species level (97% genetic identity). Analysis revealed changes in indigenous taxon-specific patterns of soil prokaryotic communities accompanying lowland rainforest transformation to jungle rubber, and intensively managed rubber and oil palm plantations. Distinct clustering of the rainforest soil communities indicated that these are different from the communities in the studied managed land use systems. The predominant bacterial taxa in all investigated soils were *Acidobacteria, Actinobacteria, Alphaproteobacteria, Betaproteobacteria*, and *Gammaproteobacteria*. Overall, the bacterial community shifted from proteobacterial groups in rainforest soils to *Acidobacteria* in managed soils. The archaeal soil communities were mainly represented by *Thaumarchaeota* and *Euryarchaeota*. Members of the Terrestrial Group and South African Gold Mine Group 1 (*Thaumarchaeota*) dominated in the rainforest and members of *Thermoplasmata* in the managed land use systems. The alpha and beta diversity of the soil prokaryotic communities was higher in managed land use systems than in rainforest. In the case of bacteria, this was related to soil characteristics such as pH value, exchangeable Ca and Fe content, C to N ratio, and extractable P content. Archaeal community composition and diversity were correlated to pH value, exchangeable Fe content, water content, and total N. The distribution of bacterial and archaeal taxa involved in biological N cycle indicated functional shifts of the cycle during conversion of rainforest to plantations.

## Introduction

Indonesia is one of the world's top producer and exporters of palm oil (Koh et al., 2011) and rubber (Marimin Darmawan et al., 2014). The continuous establishment of productive and profitable agricultural areas is accompanied by conversion of rainforest into highly productive agricultural land. This results in severe negative and irreversible effects on biodiversity and, thereby, on tropical ecosystem functions (Gibbs et al., 2010). Tropical rainforests are reckoned as important reservoirs of biodiversity (Gibson et al., 2011), which are threatened by anthropogenic demand for productive land.

Soil microbial communities contain the highest level of prokaryotic diversity of any environment, drive nearly all biogeochemical cycles in terrestrial ecosystems and participate in most nutrient transformations (Daniel, 2005; Falkowski et al., 2008; Delmont et al., 2012). It has been reported that land use and plant species as well as soil characteristics, such as pH, organic C content, and soil texture, shape soil microbial community composition and diversity (Nacke et al., 2011; Lauber et al., 2013; Pfeiffer et al., 2013). The conversion of rainforest to agricultural and plantation systems has a substantial impact on plant and animal diversity (Soares-Filho et al., 2006; Barnes et al., 2014). Despite the importance of soil microorganisms for ecosystem function, the response of microorganisms to land use change is poorly understood. Little is known on how environmental differences, e.g., changes in soil characteristics related to transformation of rainforests to rubber and oil palm plantations affect the composition, diversity and functions of soil microbial communities in general and at different spatial scales. Insights into drivers of microbial communities in tropical land use systems are limited, as an appropriate experimental design allowing robust statistical analysis and methods enabling fine taxonomic resolution are lacking in many studies.

The majority of available data on microbial communities have been collected in South and Central America such as Brazil, Ecuador, and Costa Rica (Carney et al., 2004; Rodrigues et al., 2013; Tischer et al., 2014). Studies targeting the impact of land use conversion on microbial community composition and diversity in tropical Asia are rare (Tripathi et al., 2012; Lee-Cruz et al., 2013). The published studies focused on microbial communities associated with deforestation and logging effects, and bacterial diversity in oil palm fruit compost (Liew et al., 2009; Tripathi et al., 2012; Lee-Cruz et al., 2013; McGuire et al., 2015). It has been suggested that deforestation for agricultural use alters microbial community composition in tropical regions (Tripathi et al., 2012, 2013; McGuire et al., 2015). DNA-based analysis of 16S rRNA genes indicated that soil bacteria of tropical forests are to some extent resilient to logging, but conversion to oil palm had a severe impact (Lee-Cruz et al., 2013). A study in the Brazilian Amazon rainforest showed that conversion of rainforest to pasture resulted in a strong response of soil microbial diversity, but in a manner different from plants and animals. In addition, a net loss of bacterial diversity was recorded (Rodrigues et al., 2013). Soil pH has been identified as the major driver of bacterial and archaeal diversity and community composition in the equatorial tropics (Tripathi et al., 2012, 2013). A recent study in Jambi (Indonesia) based on phospholipid fatty acid analysis showed that soil microbial biomass did not vary significantly between land use systems, although bacterial community structure changed (Krashevska et al., 2015). However, detailed information on composition and diversity of prokaryotic communities in these land use systems is still lacking.

The aim of this study was to assess the impact of agricultural demand-driven rainforest conversion to rubber and oil palm plantations on soil bacterial and archaeal community composition and diversity. This study was carried out in two contrasting landscapes with respect to soil fertility within the province Jambi of southwest Sumatra (Indonesia): Harapan with low-fertility loam Acrisol soil and Bukit Duabelas with relatively high-fertility clay Acrisol soil (Allen et al., 2015). In addition, we related changes in bacterial and archaeal composition and diversity to soil properties and abiotic and biotic factors. In this way, taxa specific-patterns and drivers of community composition associated with land use change were identified. We used large-scale amplicon-based pyrosequencing of 16S rRNA genes to assess prokaryotic community composition and diversity in the studied land use systems. Overall, we investigated the following hypotheses: (a) conversion of rainforests changes the distribution and abundance of dominant prokaryotic groups in soil, (b) species richness is highest in rainforest and lowest in managed plantations, (c) richness and diversity of prokaryotes are similar in rainforest and anthropogenically less-altered jungle rubber, and (d) rainforest conversion has a large-scale impact on the abundance of soil microbial groups and community composition by influencing soil properties and vice versa. Our study provides insights into the impacts of the rapidly expanding conversion of lowland rainforests to tree cash-crop plantations on prokaryotic soil communities.

## Materials and methods

### Sampling site description and sample recovery

**Figure 1:**
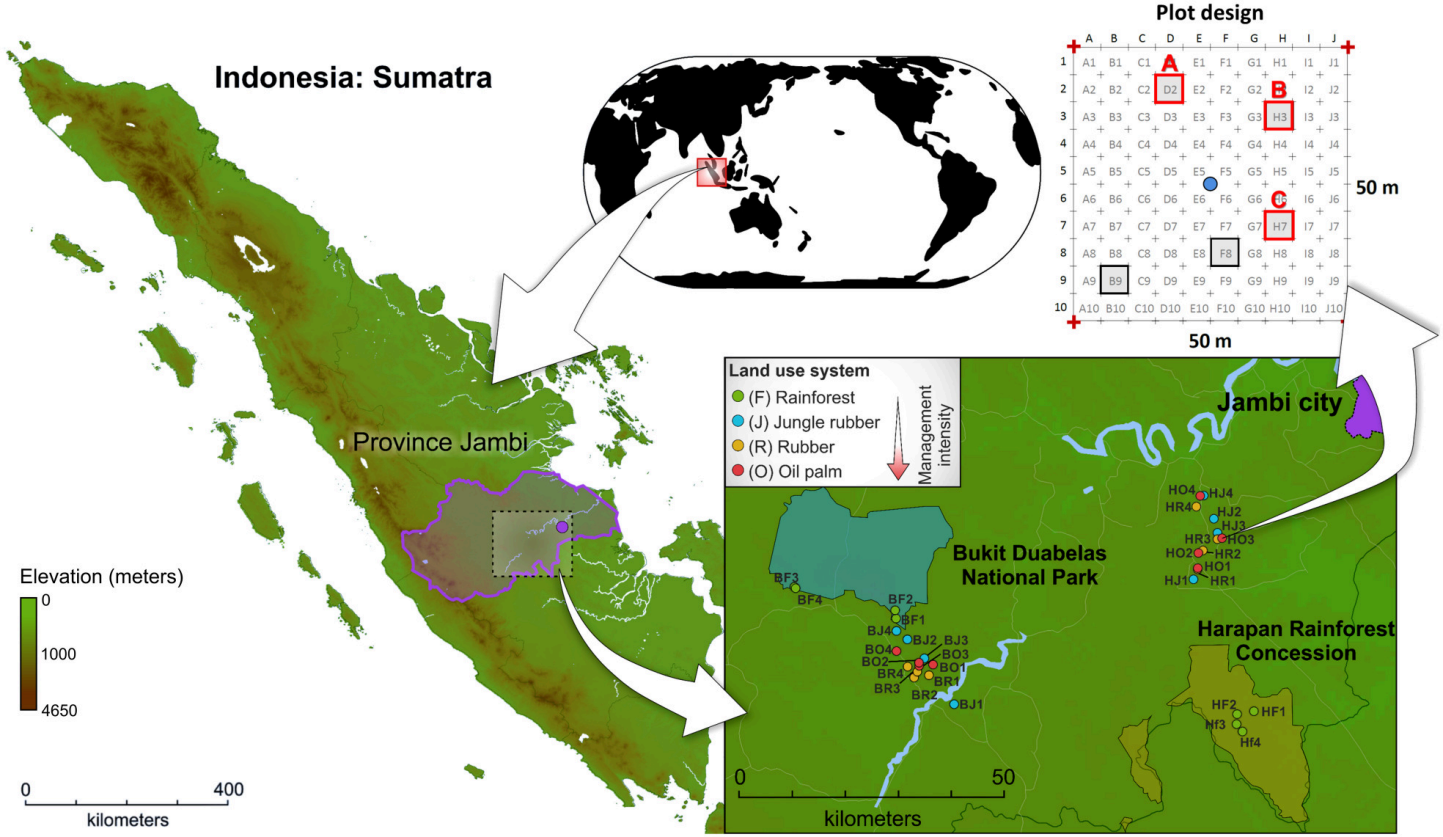
**Location of Sumatra Island (Indonesia), location of study sites within the investigated landscapes Bukit Duabelas (B) and Harapan (H), and subplot design**.

The sampling sites are located in the province Jambi of southwest Sumatra, Indonesia (Figure 1, map data was obtained from http://www.diva-gis.org/). The two landscapes, Harapan Rainforest Concession (H) and Bukit Duabelas (b), were selected for this study. Both landscapes harbor the typical land use systems in Sumatra, resulting from conversion of lowland rainforest to managed rubber and oil palm systems. In addition, suitable lowland rainforest sites (reference sites) were still present in both landscapes. Soil texture differed, with primarily loam Acrisol soils in Harapan and clay Acrisol soils in Bukit Duabelas. Within each landscape we analyzed four land use systems: secondary lowland rainforest, rubber agroforest (jungle rubber), rubber plantation, and oil palm plantation. The rainforest sites represent systems with low anthropogenic influence. Jungle rubber represents the next higher level of anthropogenic influenced land use systems. Jungle rubber is a traditional extensively managed agroforestry system, which is established by planting rubber trees into secondary rainforest. The systems with the highest anthropogenic impact are rubber and oil palm plantations, which are monocultures with high fertilizer usage and liming. The age of the rubber trees (*Hevea brasiliensis*) in jungle rubber and rubber plantation land use systems ranged from 15 to 40 and 6 to 16 years, respectively. The age of oil palm trees (*Elaeis guineensis*) in plantations varied between 8 and 15 years. The agricultural management for both plantation types included application of herbicides every 6 months and amendment of 100–300 kg ha ^−1^ yr ^−1^ inorganic NPK fertilizer in rubber plantations and 300–600 kg ha ^−1^ yr ^−1^ in oil palm plantations (for details, see Kotowska et al., 2015).

Sampling of soils was carried out from November to December 2012. The four land use systems lowland rainforest (core plots BF1-BF4 and HF1-HF4), jungle rubber (core plots BJ1-BJ4 and HJ1-HJ4), rubber plantations (core plots BR1-BR4 and HR1-HR4), and oil palm plantations (core plots BO1-BO4 and HO1-HO4) were replicated four times resulting in 32 sampling sites (for georeferences, see Table S1). Soil cores were recovered from three subplots of each plot, resulting in a total of 96 subplots. After removal of litter and root overlay, three soil cores (5–7 cm top soil, 10–20 g soil each) were taken with a soil corer and a shovel from each subplot at an average distance of 1.90 m to adjacent trees (random trees in rainforest). The samples were stored in sterile plastic bags. Subsequently, the soil samples were transported in cool boxes on ice packs to the laboratory in Indonesia within 12 h. The three soil samples per subplot were homogenized and coarse roots and stones (>5 mm) were removed. The composite samples were frozen and stored at a deep freezer (−40°C) until shipment to Germany. Samples were transported frozen (cool boxes and ice packs) to the German laboratory within approximately 25 h and stored there at −80°C until further use. Further information on sampling sites and experimental design are given in Barnes et al. (2014).

### Nucleic acid isolation and amplification of 16S rRNA genes

To analyze the prokaryotic community richness and composition soil DNA was isolated from the four land use systems by employing the PowerSoil DNA isolation kit (Dianova, Hamburg, Germany) as recommended by the manufacturer. The hypervariable regions V3 to V5 of the 16S rRNA gene were targeted in this study. The 16S rRNA gene amplicons were generated as described by Schneider et al. (2013). In brief, we employed the forward primer V3for_B (5′-CGTATCGCCTCCCTCGCGCCATCAG-MID-TA CGGRAGGCAGCAG-3′) (Liu et al., 2007) and the reverse primer V5rev_B (5′-CTATGCGCCTTGCCAGCCCGCTCAG-MID-CCGTCAATTCMTTTGAGT-3′) (Wang and Qian, 2009) for bacteria. For amplification of archaeal 16S rRNA genes, the forward primer V3for_A (5′-CGTATCGCCT CCCTCGCGCCATCAG-MID-CCCTAYGGGGYGCASCAG-3′) (Gantner et al., 2011) and the reverse primer V5rev_A (5′-CTATGCGCCTTGCCAGCCCGCTCAG-MID-GTGCTCCCCC GCCAATTCCT-3′) (Teske and Sørensen, 2008) were used. The following thermal cycling scheme was used for amplification of partial bacterial 16S rRNA genes: initial denaturation at 98°C for 5 min, 25 cycles of denaturation at 98°C for 45 s, annealing for 45 s at 65°C, and extension at 72°C for 30 s, followed by a final extension period at 72°C for 5 min. For amplification of the archaeal 16S rRNA genes, the annealing temperature was adjusted to 60°C. For two subplots (BF4 and HF3) we were unable to generate archaeal amplicons. All amplicon PCRs were performed in triplicate and pooled in equimolar amounts for sequencing. The Göttingen Genomics Laboratory determined the sequences of the 16S rRNA gene amplicons by using a 454 GS-FLX sequencer (Roche, Mannheim, Germany) and Titanium chemistry following the instructions of the manufacturer for amplicon sequencing.

### Bioinformatic analysis of 16S rRNA gene sequences

The resulting 16S rRNA gene sequences were processed and analyzed employing QIIME 1.8 (Caporaso et al., 2010). Initially, sequences shorter than 300 bp, containing unresolved nucleotides, exhibiting an average quality score lower than 25, harbor mismatches longer than 3 bp in the forward primer, or possessing homopolymers longer than 8 bp were removed with *split_libraries.py*. Additionally, we used cutadapt (Martin, 2011) with default settings for efficient reverse primer removal. Subsequently, pyrosequencing noise was removed by employing Acacia (Bragg et al., 2012) with default settings. Chimeric sequences were removed using UCHIME (Edgar et al., 2011) with Ribosomal Database Project (RDP) (Cole et al., 2014) as reference dataset (trainset10_082014_rmdup.fasta).

Operational taxonomic unit (OTU) determination was performed at a genetic divergence of 3% (species level) with *pick_open_reference_otus.py* using the Silva NR SSU 119 database as reference (Quast et al., 2013). Taxonomic classification was performed with *parallel_assign_taxonomy_blast.py* against the same database. OTU tables were created using *make_otu_table.py*. Singletons, chloroplasts, unclassified OTUs and extrinsic domain OTUs were removed from the table by employing *filter_otu_table.py*. Singletons were removed to improve comparability and avoid possible inclusion of artificial sequences (Zhou et al., 2011). Sample comparisons were performed at the same surveying effort (bacteria 6800 and archaea 2000 sequences). Diversity estimates and rarefaction curves were generated by employing *alpha_rarefaction.py*. Non-metric multidimensional scaling (NMDS) and statistical tests were performed with the vegan package (Oksanen et al., 2015) in R (R Development Core Team, 2013) and based on weighted Unifrac (Lozupone et al., 2011) distance matrixes. Significance was determined using the *envfit* function of vegan package in R (Gergs and Rothhaupt, 2015) to fit environmental vectors and factors onto the NMDS. Significance of tested variables are indicated in brackets. Profile clustering networks were constructed based on complete and subsampled OTU tables using the QIIME script *make_otu_network.py*.

### Soil characteristics

Soil parameters and properties, i.e., pH, P, N, C, C to N ratio, Al, Ca, Fe, K, Mg, Mn, Na, effective cation exchange capacity (ECEC) and base saturation for all analyzed samples were retrieved from Allen et al. (2015). Furthermore, basal respiration, microbial biomass, and soil moisture were retrieved from Krashevska et al. (2015). These data were used for statistical tests as detailed in Table S1. Data was tested for normal distribution with *shapiro.test* of stats package in R (R Development Core Team, 2013). Data that did not pass normality test (*P* < 0.05) was log transformed and normality test was repeated. Only data that passed normality test was used for further analyses. ANOVA analyses were performed with the *aov* function of stats package in R (R Development Core Team, 2013). Comparisons of land use soil characteristics were performed with Tukey's HSD (Honestly Significant Difference) by using *HSD.test* function of agricolae package in R (Mendiburu, 2015; Table S2).

### Accession numbers

The 16S rRNA gene sequences were deposited in the National Center for Biotechnology Information (NCBI) Sequence Read Archive (SRA) under accession number SRP056374.

## Results and discussion

### Study site and general soil characteristics

The study formed part of the “Ecological and Socioeconomic Functions of Tropical Lowland Rainforest Transformation Systems” (EFForTS) collaborative research center, which analyzes various aspects of tropical lowland rainforest conversion to agricultural systems in Indonesia, including the impact on aboveground and belowground biodiversity, soil fertility, nutrient fluxes and greenhouse gas emissions as well as the economic, social, cultural and political dimensions (Barnes et al., 2014; Guillaume et al., 2015; Krashevska et al., 2015). We analyzed an agricultural management gradient with increasing intensity from jungle rubber over rubber plantations to oil palm plantations in two landscapes (Bukit Duabelas and Harapan). Soils from lowland rainforest sites served as reference. The soils comprised relatively fertile, clay loam Acrisol soil in Bukit Duabelas and less fertile, loam Acrisol soil in Harapan (Table S1).

Although the investigated systems were non-artificial, the soil parameters showed clear patterns for the land use systems (Table S1 and Figure S1). The analyses of soil characteristics between land use systems by ANOVA and Tukey's HSD showed that the soils of the analyzed land use types did not vary significantly in N, C, basal respiration, microbial biomass, moisture and silt content (Table S2). Significant differences between land use types were observed for pH values, P content and clay content (Table S2). Soil pH increased slightly from an average of 4.21 to 4.45 from rainforest to oil palm plantations in both landscapes, which likely is due to liming. Bioavailable micro- and macroelements, i.e., Mn, Na, C, Ca, Fe, Mg, and N had an overall higher concentration in Bukit Duabelas soils than in Harapan soils (see Table S1 and Figure S2). Organic carbon was generally lower in the managed systems rubber and oil palm. Soil moisture was roughly three-fold higher in Bukit Duabelas than in Harapan (Table S1).

### Effect of rainforest transformation on bacterial diversity and community composition

DNA from each subplot was used for amplification of the V3–V5 hypervariable region of the bacterial 16S rRNA gene. Sequencing and quality filtering resulted in 1,367,923 high-quality 16S rRNA gene sequences from all subplots. After removal of singletons, the dataset comprised 16,413 OTUs at 97% genetic identity. After subsampling (6800 sequences per sample), the average number of OTUs per subplot was 1160 ± 245 ranging from 604 (BF4b) to 1825 (HO2b) OTUs (Table S3).

Soil bacterial diversity significantly responded to land use change from rainforest to plantations (*P* = 0.001, *r*^2^ = 0.7875). Richness and diversity incrementally rose with increasing management intensity from rainforest to oil palm plantations (rainforest < jungle rubber < rubber plantations < oil palm plantations, see Figure S3). This was different from the responses of animals and plant diversity to land use conversion, which showed the opposite trend (Barnes et al., 2014). Accordingly, Shannon indices of diversity of the bacterial communities differed between rainforest in relation to the managed land use systems (*P* = 0.001, *r*^2^ = 0.5956, Table S3). Phylogenetic diversity (PD, *P* = 0.001, *r*^2^ = 0.7349) showed a similar trend, indicating highest diversity in the managed land use systems (Table S3). Rarefaction curves showed slight saturation at the same surveying effort, which indicates that the datasets covered all main bacterial groups thriving in the investigated land use systems (Figure S3). In addition, the bacterial diversity slightly varied between the two studied landscapes. In all land use systems the diversity was slightly higher in the Harapan than in the Bukit Duabelas region. Landscape alone had no significant effect on bacterial community composition (*P* > 0.8).

**Figure 2:**
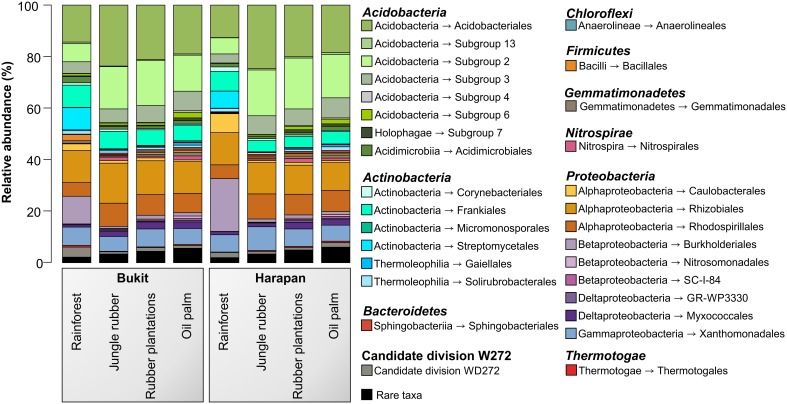
**Bacterial community composition based on relative abundances separated by landscape and land use system**. The results of all analyzed samples of each land use system in a landscape were summarized (for individual results, see bacterial OTU table Data Sheet 1).

The analysis of the bacterial community composition and abundance of taxa within the different land use systems revealed the main bacterial groups thriving in the studied systems and their different abundances (Figure 2). The composition of soil bacterial communities varied between the different land use systems, but was very similar within a land use system. The most abundant phyla in all samples were *Acidobacteria* (42.7%) followed by *Proteobacteria* (37.7%), *Actinobacteria* (12.6%), and to a lesser degree Candidate Division WD272 (1.6%), *Firmicutes* (1.2%), *Chloroflexi* (1.0%), *Nitrospirae* (0.9%), *Gemmatimonadetes* (0.8%), and *Bacteroidetes* (0.6%). These bacterial phyla and Candidate Division are common in a variety of different soils, including non-tropical forest, grassland and agricultural soils (da C Jesus et al., 2009; Nacke et al., 2011; Lauber et al., 2013; Tripathi et al., 2014).

**Figure 3:**
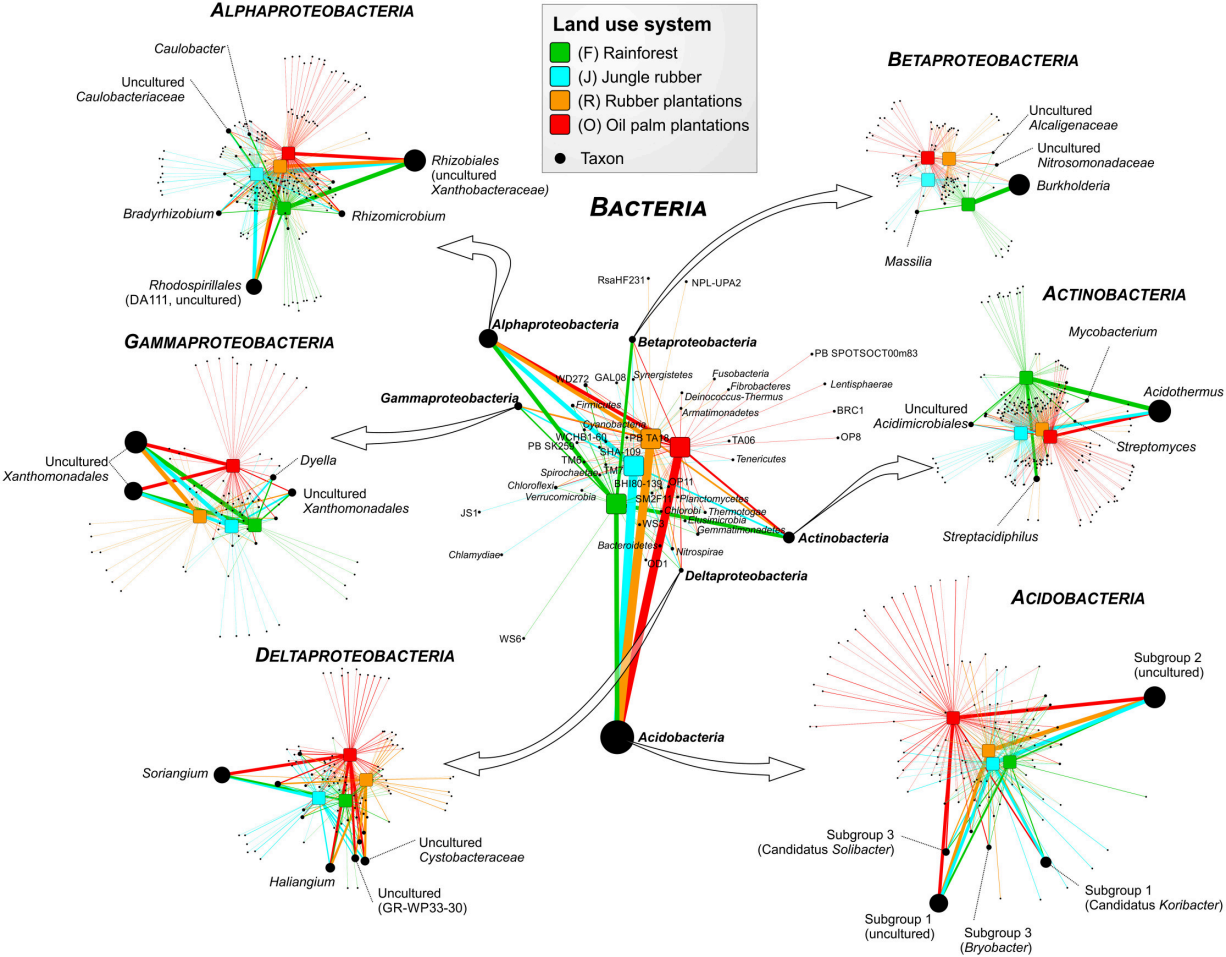
**Profile clustering network analysis of bacterial phyla and proteobacterial classes in the land use systems and detailed networks of the most abundant classes**. The width of the node connecting lines defines the mean relative abundance of the bacterial taxon in the corresponding land use system. The size of each node is proportional to the mean taxon abundance between all land use systems.

Soil bacterial communities in rainforest sites of both studied landscapes were very similar (Figure 2). The same was found for the communities in the other land use systems of both landscapes, indicating a management-specific shift of the bacterial community structure. Differences between the studied land use systems were mainly encountered at higher taxonomic resolution (Figure 3).

A comparison of *Acidobacteria* at order level revealed that the relative abundances of subgroups 2, 4, 5, 6, and 7 were higher in soils of the managed systems than in rainforest soils. *Acidobacteriales* showed the highest abundance in jungle rubber, followed by rubber, oil palm plantations, and rainforest. Additionally, our results indicate a general pH optimum for most of the encountered acidobacterial groups at a pH of approximately 4.4 (data not shown). Within the *Actinobacteria, Acidimicrobiales* exhibited significant higher relative abundances in rainforest and oil palm than in jungle rubber and rubber plantations. *Frankiales, Streptomycetales*, and *Corynebacteriales* were more abundant in rainforests than in the managed land use systems.

Among *Alphaproteobacteria, Rhizobiales* were abundant in all land use systems, whereas *Caulobacterales* revealed a higher abundance in rainforest than in the managed land use systems. *Rhodospirillales* showed the opposite trend. The betaproteobacterial *Burkoholderiales* (i.e., *Burkholderia tropica*; Reis et al., 2004) were more abundant in rainforest (up to 20.5% in Harapan rainforest soils) compared to the managed systems (< 1.5%). *Burkholderia* seems to be one of the key bacterial groups for nitrogen-fixation in rainforest soils, since several species are known as plant-associated nitrogen-fixing bacteria (Estrada-De Los Santos et al., 2001). The decrease of *Burkholderia* species followed the increased use of fertilizer in the plantation systems. The increase of bioavailable nitrogen through fertilization in intensively managed soils provides other bacterial taxa with improved growth conditions. This leads to a decrease of nitrogen-fixing bacteria. This observation is confirmed by studies on N availability in the investigated land use systems, which decreased along the described land use gradient (Allen et al., 2015). Members of the *Nitrosomonadales* and SC-I-84 were almost absent in rainforest soils, but showed an increasing abundance (up to 1%) along the gradient from unmanaged rainforest to intensively managed oil palm plantations. The increased abundance of Nitrosomonadales, which are known as ammonia-oxidizing bacteria (Shen et al., 2012), also followed the increase of fertilizer treatment in the intensively managed plantations.

The main representatives of the *Deltaproteobacteria* were the fungi-like *Myxococcales* (*Soriangium* and *Haliangium*) and GR-WP33-30, which were slightly more abundant in the managed soils. The increased abundance of myxobacteria in managed systems is another indication of increased anthropogenic influence (fertilization, dung) within managed land use systems (Brenner et al., 2005). Within the *Gammaproteobacteria*, the *Xanthomonadales* were the predominant order, however, their abundance did not differ significantly between the analyzed land use systems. Interestingly, conversion to fertilized soils in plantations did not increase the abundance of potentially harmful *Gammaproteobacteria* in soils, as recently shown for Mexican agricultural soils fertilized with wastewater (Broszat et al., 2014).

Despite the higher diversity in the managed land use systems, several phylogenetic groups were specific for the rainforest soils and not present in other soils, including several genera within the *Alphaproteobacteria* and *Actinobacteria*. Thus, conversion of rainforests leads to a loss of rainforest endemic bacterial groups. However, several taxa thrive only in managed soils, which is likely due to higher pH and higher nutrient availability derived from fertilization. Other effects can also be accounted for the occurrence of certain taxa, i.e., the abundance of photosynthetic *Cyanobacteria* and *Rhodospirillales* increased in oil palm plantations. This is presumably due to the more open canopy (monocultures) in oil palm plantations compared to rainforest, which results in higher light levels on the ground supporting growth of photosynthetically active bacterial groups. Other examples were members of the *Bacteroidetes*, i.e., uncultured *Chitinophagaceae*, which were slightly more abundant in managed systems. Members of this family are known as chitin degraders and the higher abundance might be linked to an increase in fungal abundance in managed systems (jungle rubber and rubber plantations), as shown by Krashevska et al. (2015).

### Influence of soil attributes on prokaryotic communities

**Figure 4:**
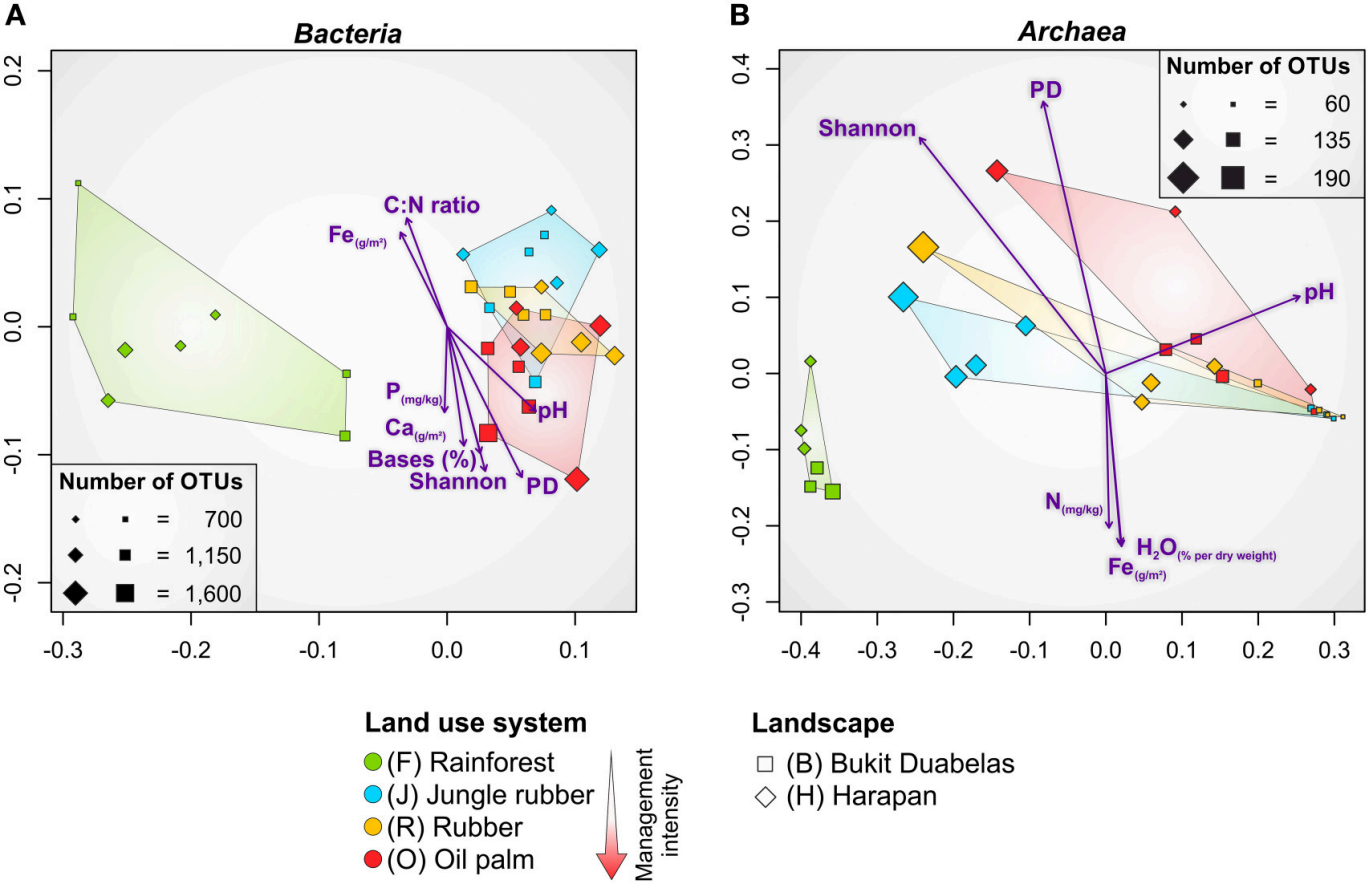
**Non-metric multidimensional scaling (NMDS) of bacterial (A) and archaeal (B) community composition in all core plots of the land use systems rainforest, jungle rubber, rubber and oil palm based on weighted Unifrac (Lozupone et al., 2011) distance matrices**. Significant correlations of environmental parameters and diversity metrices (Shannon, PD) to community composition are shown by purple arrows (*P* ≤ 0.045). Size of plots (squares and diamonds) corresponds to number of observed OTUs at species level (97% genetic similarity). Bases, base saturation; PD, phylogenetic diversity.

NMDS confirmed that the dissimilarities in community composition were driven by conversion of rainforest to agriculturally managed systems (Figure 4A). Soil bacterial communities in managed systems such as rubber and oil palm plantations clearly separated from those of rainforest soils. The rainforest communities formed a distinct cluster. From all measured biotic and abiotic soil parameters, environmental parameters, such as soil pH and base saturation (pH, *P* = 0.001, *r*^2^ = 0.4465; base saturation, *P* = 0.001, *r*^2^ = 0.4573), and to a lesser extent exchangeable Ca and Fe content, C to N ratio, and extractable P content correlated with bacterial community composition (Ca, *P* = 0.003, *r*^2^ = 0.3861; Fe, *P* = 0.011, *r*^2^ = 0.2957; C to N ratio, *P* = 0.003, *r*^2^ = 0.3078; P, *P* = 0.042, *r*^2^ = 0.1801). It is known that soil pH is one of the major drivers of bacterial community structure (Lauber et al., 2009; Rousk et al., 2010; Nacke et al., 2011), but in the here analyzed landscapes base saturation exhibited a higher impact. Since base saturation is closely connected to soil pH, this effect was expected. The exchangeable Ca content generally increased from rainforest to oil palm plantation due to liming. The C to N ratio, a predictor for nitrogen availability, was lower in managed soils due to fertilizer usage. In addition to plants, also specialized bacterial taxa use the supplied nitrogen fertilizer. This was indicated by the increase in ammonia-oxidizing bacteria (*Nitrosomonadales*) in plantations. Exchangeable Fe was higher in rainforest soils possibly due to the higher acidity. Thus, fertilization temporarily increased bacterial diversity, however, recovery potential of managed soils was not investigated in this study. To analyze the recovery potential of agriculturally used rainforest soils the absence of fertilization for longer time periods would be a requirement.

As recorded for bacterial communities, NMDS analysis of the soil archaeal community composition also showed that the dissimilarities were driven by conversion of rainforest to managed systems (Figure 4B). The soil archaeal communities in managed land use systems also clearly separated from that in rainforest soils and showed distinct clustering. Soil pH was also related to archaeal community structure but compared to bacteria to a lesser extent (pH, *P* = 0.002, *r*^2^ = 0.3768). Additionally, less significant association of archaeal communities was observed for Fe content (*P* = 0.020, *r*^2^ = 0.2599), water content (*P* = 0.029, *r*^2^ = 0.2526), and total N (*P* = 0.034, *r*^2^ = 0.2058). This suggests a negative correlation of archaeal taxa with an increase of soil moisture, Fe and N content, indicating the preference of certain archaea for habitats with harsher (more extreme) conditions such as low pH values, low water content, and limited availability of nutrients and energy sources (Chaban et al., 2006).

### Effect of rainforest transformation on archaeal diversity and community composition

*Archaea* are important members of soil prokaryotic communities, but constitute on average only about 2% of the prokaryotic soil community (Bates et al., 2011). The entire curated dataset of all analyzed plots contained 438,500 archaeal 16S rRNA gene sequences and comprised 1679 OTUs at species level (97% genetic identity). The average number of OTUs per sample was 113 ± 41 and ranged from 53 (BR3a) to 234 (HJ2a) OTUs (Table S4). Archaeal diversity was generally higher in the Harapan region compared to the corresponding land use systems of the Bukit Duabelas region. The archaeal communities of Harapan soils followed roughly the same trend as the bacterial communities and diversity increased in the managed systems (Figure S4 and Table S4). The communities in the soils from the Bukit Duabelas region revealed a different behavior, as the archaeal communities in both rubber land use systems showed a lower diversity compared to rainforest and oil palm plantations. This difference might be linked to the lower humidity and reduced availability of nutrients and energy sources in the Harapan soils (see Figure 4B and Table S1).

**Figure 5:**
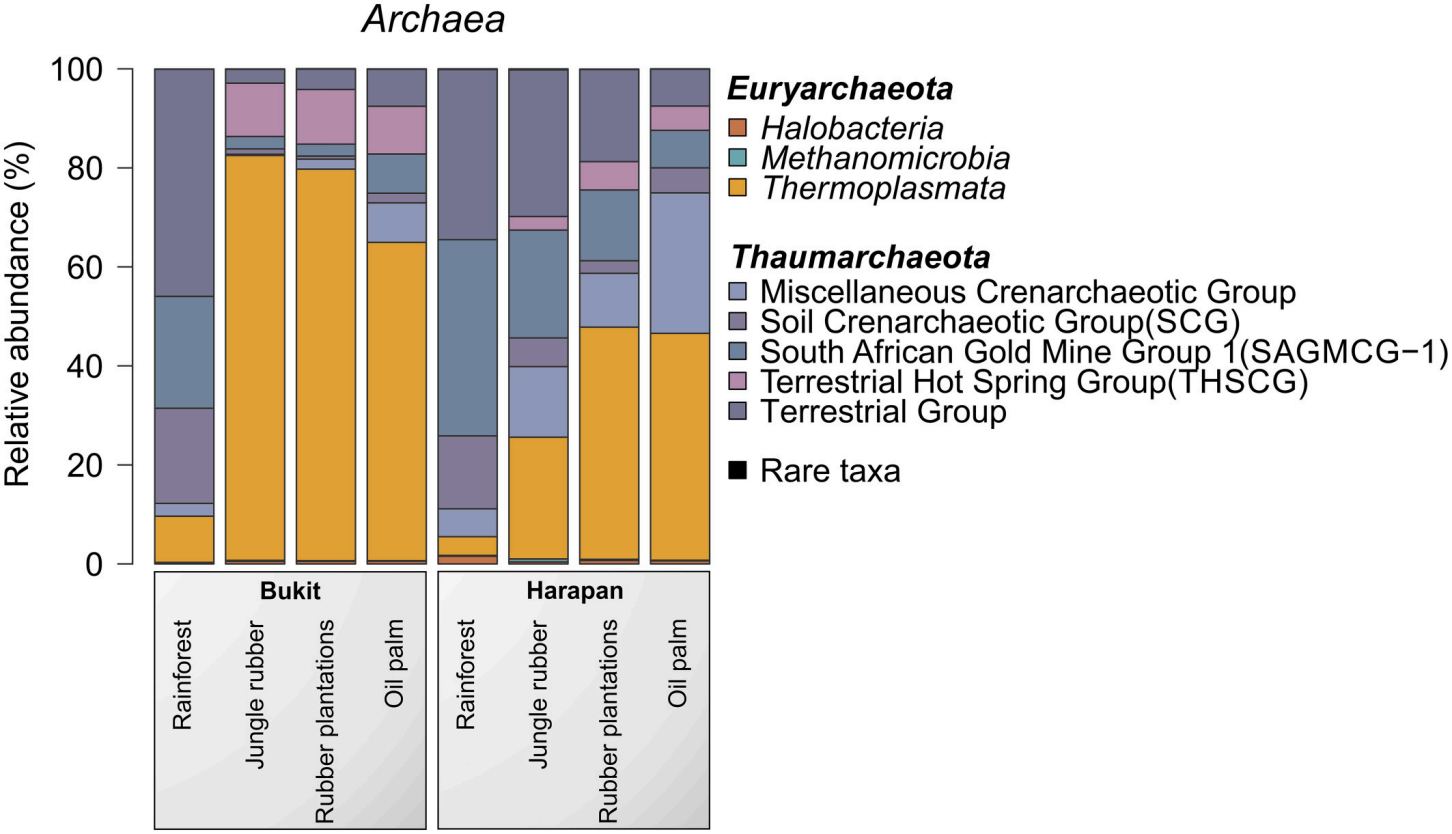
**Archaeal community composition based on relative abundances separated by landscape and land use system**. The results of all analyzed samples of a land use system in a landscape were summarized (for individual results, see archaeal OTU table Data Sheet 2).

The most abundant archaeal phyla in all samples were *Thaumarchaeota* (54.6%) and *Euryarchaeota* (45.3%). *Crenarchaeota* were present in all land use systems but only in very low amounts (< 0.1%). Archaeal diversity varied among landscapes and land use systems. A distinct shift from *Thaumarchaeota* to *Euryarchaeota* was observed from rainforest to the managed land use system in both landscapes. This effect was more pronounced in the Bukit Duabelas landscape (Figure 5).

*Thaumarchaeota* contain important taxa involved in the soil N cycle such as *Nitrososphaera* species. *Nitrososphaera* species have been recently isolated and characterized. Members of these species are involved in nitrification by performing oxidation of ammonia (Spang et al., 2012). The abundance of uncultured members of Candidatus *Nitrososphaera*, which is part of Soil Crenarchaeotic Group (SCG), increased almost five-fold from rainforest to oil palm (0.2–0.9%). Thus, the abundance of nitrogen-oxidizing *Thaumarchaeota* rose with increasing fertilization performed in the managed plantations (rubber and oil palm plantations). This trend was coupled with a concurrent increase in ammonia-oxidizing bacteria (*Nitrosomonadales*, see above). This suggested interactions between these groups and functional changes in the biological N cycle during transformation of rainforest into plantations (Bates et al., 2011).

**Figure 6:**
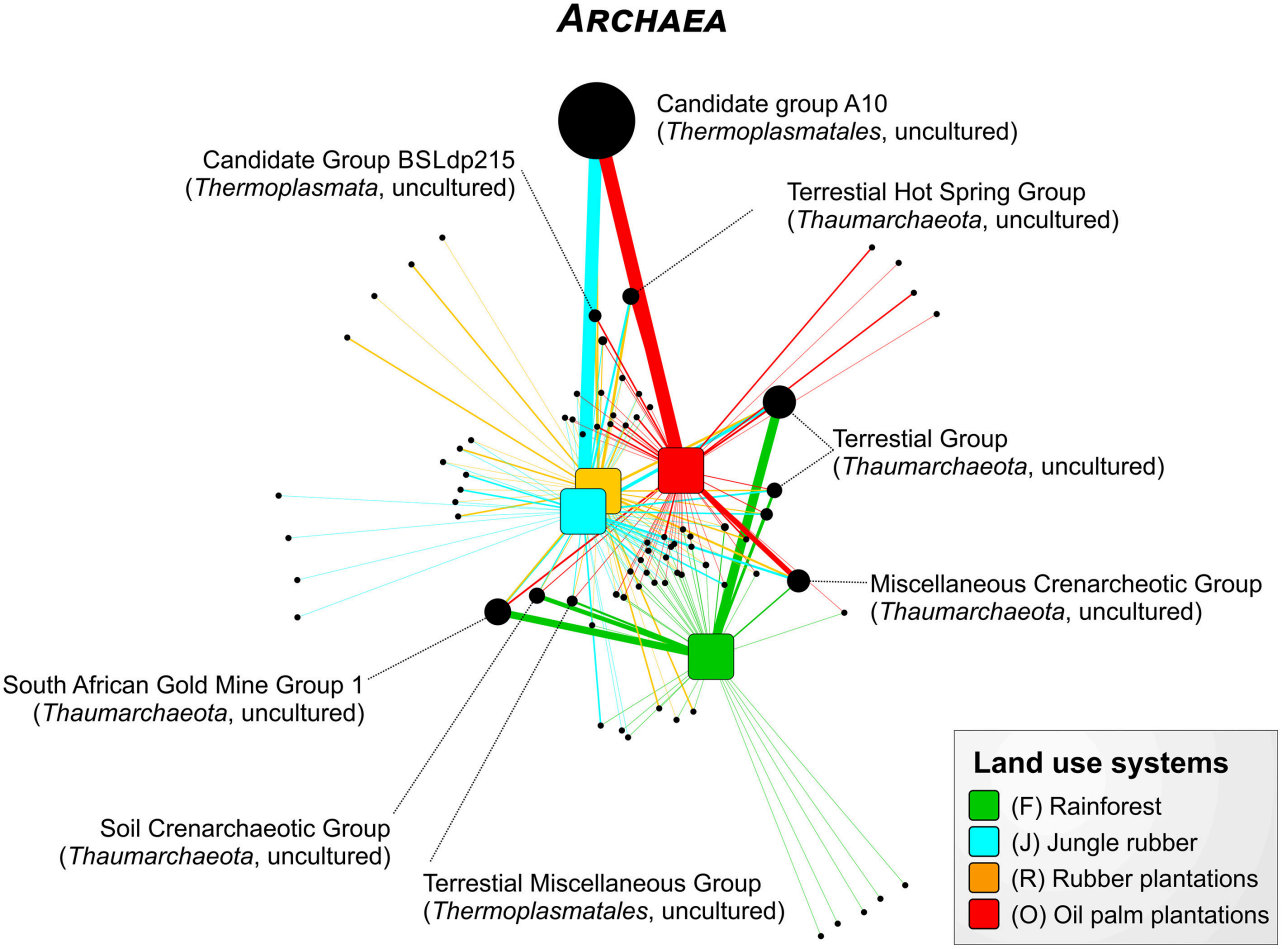
**Profile clustering network analysis of the archaeal taxa composition in the land use systems**. The width of the node connecting lines defines the mean relative abundance of the archaeal taxon in the according land use system. The size of each node is proportional to the mean taxon abundance between all land use systems.

For the Bukit Duabelas landscape soils, the *Thaumarchaeota* and *Euryarchaeota* showed distinct differences in relative abundances between rainforest and the other land use systems (Figure 6). A comparison of *Thaumarchaeota* at order level revealed that the relative abundance of the Terrestrial Group was higher in rainforest than in managed soils. This also applied for subgroups SAGMGC-1 and SCG whereas Terrestrial Hot Spring Group (THSCG) and Miscellanous Crenarchaeotic Group (MCG) increased in managed soils. Members of SAGMGC-1 include ammonia-oxidizing archaea and prefer low pH environments (Auguet and Casamayor, 2013). This was also observed for Harapan soils, but in comparison to Bukit Duabelas soils the change from *Thaumarchaeota* to *Euryarchaeota* was less pronounced. Additionally, the THSCG only slightly increased in abundance from rainforest to oil palm. The MCG was more abundant in Harapan than in Bukit Duabelas soils. The predominant *Thermoplasmatales* was Candidate Group A10, which was absent from rainforest soils. Interestingly, the dominant OTUs of this group have relatives in extreme environments like sediments of thermoacidophilic volcanic springs (Eme et al., 2013; Wemheuer et al., 2013), indicating that the effect of pH outweighed the influence of temperature. Unfortunately, little is known on the metabolic potential of the other archaeal taxa and possible traits cannot be deduced currently.

## Conclusion

According to our hypothesis (a), the conversion of rainforest to managed systems significantly impacts soil prokaryotic (bacteria and archaea) community structure, diversity and correspondingly, functional traits. Distinct clustering of the rainforest soil communities indicated that these are different from the communities in the studied managed land use systems.

The soil communities in the low-intensity managed jungle rubber system were more closely related to that in the plantation systems than to that in rainforest. This is in contrast to our hypothesis (c), that prokaryotes in rainforest and jungle rubber sites are more similar. However, jungle rubber sites are considered as the intermediate system between rainforest and plantation sites. Additionally, the jungle rubber soil communities showed greater similarity to those in rubber plantations than to those in rainforest soils, indicating a management and possibly tree species impact.

We recorded an increase of soil prokaryotic diversity from rainforest to oil palm plantations. This is in contrast to animals, fungi, and plants and our hypothesis (b) stating that rainforest conversion to agriculturally managed systems negatively impacts prokaryotic diversity. Several bacterial and archaeal taxa were specific for rainforest soils and not present in the other land use systems. Thus, despite an increase in diversity in the managed systems the conversion of rainforests to managed systems leads to a net loss in prokaryotic biodiversity, which is coupled to a loss of traits (Rodrigues et al., 2013). The long-term effect of this loss is not known and has to be determined in long-term studies, e.g., analysis of the recovery potential of soil prokaryotic communities after reforestation or in the absence of management treatments like fertilization. In particular, it is unknown whether the rainforest endemic taxa are truly locally extinct or still present in the managed land use systems, but in such low abundance that they were not detected by our surveying effort. However, saturation of the rarefaction curves indicated that we provided a comprehensive survey of soil prokaryotic communities in the studied systems. Nevertheless, we also observed unique prokaryotic taxa in the other land use systems, resulting in highest diversity in oil palm plantations. It will be awarding to evaluate if traits from endemic species exist in other taxa that are present in the soil communities of other land use systems, as redundancy for many biogeochemical and other gene families across soil microbial groups exists.

In accordance with our hypothesis (d), the conversion of rainforests resulted in significant changes of the prokaryotic community composition. The reduction of nitrogen-fixing bacterial community members in plantations due to fertilizer usage may negatively impact soil fertility on the long term. In addition, it is indicated that treatment-induced changes of soil characteristics, especially vigorous fertilization in oil palm plantations, support prokaryotic diversity.

In the near future, we will focus on the analysis of temporal and treatment-induced (e.g., pesticide treatment) changes of the soil microbial community structures and their functions along the different land use systems in the tropics. In addition, interaction networks between different prokaryotic functional groups and other soil organisms including fungi will be investigated to deepen our understanding of global impacts of large-scale rainforest transformation on soil ecosystem functions.

## Author contributions

RD designed and conceived the study; Soil sampling for prokaryotic community analysis was performed by ME, MW, and AM; DS, ME, KA, SK, VK, and MH carried out the field and laboratory work; DS, KA, and VK prepared and analyzed the data; all authors interpreted the results and wrote the paper.

### Conflict of interest statement

The authors declare that the research was conducted in the absence of any commercial or financial relationships that could be construed as a potential conflict of interest.

## Supplementary material

Data Sheet 1 **Bacterial OTU table at subplot level including taxonomic information**.

Data Sheet 2 **Archaeal OTU table at subplot level including taxonomic information**.

Table S1 **Soil characteristics and georeferences of the analyzed plots**.

Table S2 **Statistical analyses of soil characteristics**.

Table S3 **Observed bacterial OTUs and diversity at subplot level with summaries at plot level**.

Table S4 **Observed archaeal OTUs and diversity at subplot level with summaries at plot level**.

Figure S1 **Box plots of soil characteristics summarized by land use system**.

Figure S2 **Box plots of soil characteristics summarized by landscape and land use system**.

Figure S3 **Rarefaction analyses of the bacterial diversity of the two analyzed landscapes and four land use systems**.

Figure S4 **Rarefaction analyses of the archaeal diversity of the two analyzed landscapes and four land use systems**.

## References

[B1] Allen K. Corre M. D. Tjoa A. Veldkamp E. (2015). Soil nitrogen-cycling responses to conversion of lowland forests to oil palm and rubber plantations in Sumatra, Indonesia. PLoS ONE 10:e0133325. 10.1371/journal.pone.0133325 26222690 PMC4519237

[B2] Auguet J. C. Casamayor E. O. (2013). Partitioning of *Thaumarchaeota* populations along environmental gradients in high mountain lakes. FEMS Microbiol. Ecol. 84, 154–164. 10.1111/1574-6941.12047 23176712

[B3] Barnes A. D. Jochum M. Mumme S. Haneda N. F. Farajallah A. Widarto T. H.. (2014). Consequences of tropical land use for multitrophic biodiversity and ecosystem functioning. Nat. Commun. 5:5351. 10.1038/ncomms6351 25350947 PMC4220457

[B4] Bates S. T. Berg-Lyons D. Caporaso J. G. Walters W. A. Knight R. Fierer N. (2011). Examining the global distribution of dominant archaeal populations in soil. ISME J. 5, 908–917. 10.1038/ismej.2010.171 21085198 PMC3105767

[B5] Bragg L. Stone G. Imelfort M. Hugenholtz P. Tyson G. W. (2012). Fast, accurate error-correction of amplicon pyrosequences using Acacia. Nat. Methods 9, 425–426. 10.1038/nmeth.1990 22543370

[B6] Brenner D. J. Krieg N. R. Staley J. T. Garrity G. M. (2005). Bergey's Manual of Systematic Bacteriology, Vol. 2, Part C. New York, NY: Springer.

[B7] Broszat M. Nacke H. Blasi R. Siebe C. Huebner J. Daniel R.. (2014). Wastewater irrigation increases the abundance of potentially harmful *Gammaproteobacteria* in soils in Mezquital Valley, Mexico. Appl. Environ. Microbiol. 80, 5282–5291. 10.1128/AEM.01295-14 24951788 PMC4136100

[B8] Caporaso J. G. Kuczynski J. Stombaugh J. Bittinger K. Bushman F. D. Costello E. K.. (2010). QIIME allows analysis of high-throughput community sequencing data. Nat. Methods 7, 335–336. 10.1038/nmeth.f.303 20383131 PMC3156573

[B9] Carney K. M. Matson P. A. Bohannan B. J. M. (2004). Diversity and composition of tropical soil nitrifiers across a plant diversity gradient and among land-use types. Ecol. Lett. 7, 684–694. 10.1111/j.1461-0248.2004.00628.x

[B10] Chaban B. Ng S. Y. Jarrell K. F. (2006). Archaeal habitats–from the extreme to the ordinary. Can. J. Microbiol. 52, 73–116. 10.1139/w05-147 16541146

[B11] Cole J. R. Wang Q. Fish J. A. Chai B. McGarrell D. M. Sun Y.. (2014). Ribosomal Database Project: data and tools for high throughput rRNA analysis. Nucleic Acids Res. 42, D633–D642. 10.1093/nar/gkt1244 24288368 PMC3965039

[B12] da C Jesus E. Marsh T. L. Tiedje J. M. de S Moreira F. M. (2009). Changes in land use alter the structure of bacterial communities in Western Amazon soils. ISME J. 3, 1004–1011. 10.1038/ismej.2009.47 19440233

[B13] Daniel R. (2005). The metagenomics of soil. Nat. Rev. Microbiol. 3, 470–478. 10.1038/nrmicro1160 15931165

[B14] Delmont T. O. Prestat E. Keegan K. P. Faubladier M. Robe P. Clark I. M.. (2012). Structure, fluctuation and magnitude of a natural grassland soil metagenome. ISME J. 6, 1677–1687. 10.1038/ismej.2011.197 22297556 PMC3498926

[B15] Edgar R. C. Haas B. J. Clemente J. C. Quince C. Knight R. (2011). UCHIME improves sensitivity and speed of chimera detection. Bioinformatics 27, 2194–2200. 10.1093/bioinformatics/btr381 21700674 PMC3150044

[B16] Eme L. Reigstad L. J. Spang A. Lanzén A. Weinmaier T. Rattei T.. (2013). Metagenomics of Kamchatkan hot spring filaments reveal two new major (hyper)thermophilic lineages related to *Thaumarchaeota*. Res. Microbiol. 164, 425–438. 10.1016/j.resmic.2013.02.006 23470515

[B17] Estrada-De Los Santos P. Bustillos-Cristales R. Caballero-Mellado J. (2001). Burkholderia, a genus rich in plant-associated nitrogen fixers with wide environmental and geographic distribution. Appl. Environ. Microbiol. 67, 2790–2798. 10.1128/AEM.67.6.2790-2798.2001 11375196 PMC92940

[B18] Falkowski P. G. Fenchel T. Delong E. F. (2008). The microbial engines that drive Earth's biogeochemical cycles. Science 320, 1034–1039. 10.1126/science.1153213 18497287

[B19] Gantner S. Andersson A. F. Alonso-Sáez L. Bertilsson S. (2011). Novel primers for 16S rRNA-based archaeal community analyses in environmental samples. J. Microbiol. Methods 84, 12–18. 10.1016/j.mimet.2010.10.001 20940022

[B20] Gergs R. Rothhaupt K.-O. (2015). Invasive species as driving factors for the structure of benthic communities in Lake Constance, Germany. Hydrobiologia 746, 245–254. 10.1007/s10750-014-1931-4

[B21] Gibbs H. K. Ruesch A. S. Achard F. Clayton M. K. Holmgren P. Ramankutty N.. (2010). Tropical forests were the primary sources of new agricultural land in the 1980s and 1990s. Proc. Natl. Acad. Sci. U.S.A. 107, 16732–16737. 10.1073/pnas.0910275107 20807750 PMC2944736

[B22] Gibson L. Lee T. M. Koh L. P. Brook B. W. Gardner T. A. Barlow J.. (2011). Primary forests are irreplaceable for sustaining tropical biodiversity. Nature 478, 378–381. 10.1038/nature10425 21918513

[B23] Guillaume T. Damris M. Kuzyakov Y. (2015). Losses of soil carbon by converting tropical forest to plantations: erosion and decomposition estimated by delta C. Glob. Chang Biol. 21, 3548–3560. 10.1111/gcb.12907 25707391

[B24] Koh L. P. Miettinen J. Liew S. C. Ghazoul J. (2011). Remotely sensed evidence of tropical peatland conversion to oil palm. Proc. Natl. Acad. Sci. U.S.A. 108, 5127–5132. 10.1073/pnas.1018776108 21383161 PMC3064377

[B25] Kotowska M. M. Leuschner C. Triadiati T. Meriem S. Hertel D. (2015). Quantifying above- and belowground biomass carbon loss with forest conversion in tropical lowlands of Sumatra (Indonesia). Glob. Chang. Biol. 21, 3620–3634. 10.1111/gcb.12979 25980371

[B26] Krashevska V. Klarner B. Widyastuti R. Maraun M. Scheu S. (2015). Impact of tropical lowland rainforest conversion into rubber and oil palm plantations on soil microbial communities. Biol. Fertil. Soils 51, 697–705. 10.1007/s00374-015-1021-4

[B27] Lauber C. L. Hamady M. Knight R. Fierer N. (2009). Pyrosequencing-based assessment of soil pH as a predictor of soil bacterial community structure at the continental scale. Appl. Environ. Microbiol. 75, 5111–5120. 10.1128/AEM.00335-09 19502440 PMC2725504

[B28] Lauber C. L. Ramirez K. S. Aanderud Z. Lennon J. Fierer N. (2013). Temporal variability in soil microbial communities across land-use types. ISME J. 7, 1641–1650. 10.1038/ismej.2013.50 23552625 PMC3721119

[B29] Lee-Cruz L. Edwards D. P. Tripathi B. M. Adams J. M. (2013). Impact of logging and forest conversion to oil palm plantations on soil bacterial communities in Borneo. Appl. Environ. Microbiol. 79, 7290–7297. 10.1128/AEM.02541-13 24056463 PMC3837752

[B30] Liew P. W. Jong B. C. Goh C. M. Ahmad M. (2009). Bacterial diversity associated with empty oil palm fruit bunch compost as revealed by cultivation-independent analyses of PCR-amplified 16S rRNA genes. J. Gen. Appl. Microbiol. 55, 233–240. 10.2323/jgam.55.233 19590151

[B31] Liu Z. Lozupone C. Hamady M. Bushman F. D. Knight R. (2007). Short pyrosequencing reads suffice for accurate microbial community analysis. Nucleic Acids Res. 35, e120. 10.1093/nar/gkm541 17881377 PMC2094085

[B32] Lozupone C. Lladser M. E. Knights D. Stombaugh J. Knight R. (2011). UniFrac: an effective distance metric for microbial community comparison. ISME J. 5, 169–172. 10.1038/ismej.2010.133 20827291 PMC3105689

[B33] Marimin Darmawan M. A. Machfud Islam Fajar Putra M. P. Wiguna B. (2014). Value chain analysis for green productivity improvement in the natural rubber supply chain: a case study. J. Clean. Prod. 85, 201–211. 10.1016/j.jclepro.2014.01.098

[B34] Martin M. (2011). Cutadapt removes adapter sequences from high-throughput sequencing reads. EMBnet. J. 17, 10–12. 10.14806/ej.17.1.200

[B35] McGuire K. L. D'Angelo H. Brearley F. Q. Gedallovich S. M. Babar N. Yang N.. (2015). Responses of soil fungi to logging and oil palm agriculture in southeast asian tropical forests. Microb. Ecol. 69, 733–747. 10.1007/s00248-014-0468-4 25149283

[B36] Mendiburu F. D. (2015). agricolae: Statistical Procedures for Agricultural Research. R Package Version 1.2-3. Available online at: https://cran.r-project.org/web/packages/agricolae/index.html

[B37] Nacke H. Thurmer A. Wollherr A. Will C. Hodac L. Herold N.. (2011). Pyrosequencing-based assessment of bacterial community structure along different management types in German forest and grassland soils. PLoS ONE 6:e17000. 10.1371/journal.pone.0017000 21359220 PMC3040199

[B38] Oksanen J. Blanchet F. G. Kindt R. Legendre P. Minchin P. R. O'Hara R. B. (2015). vegan: Community Ecology Package. R Package Version 2.2-1. Available online at: https://cran.r-project.org/web/packages/vegan/index.html

[B39] Pfeiffer B. Fender A.-C. Lasota S. Hertel D. Jungkunst H. F. Daniel R. (2013). Leaf litter is the main driver for changes in bacterial community structures in the rhizosphere of ash and beech. Agric. Ecosyst. Environ. Appl. Soil Ecol. 72, 150–160. 10.1016/j.apsoil.2013.06.008

[B40] Quast C. Pruesse E. Yilmaz P. Gerken J. Schweer T. Yarza P.. (2013). The SILVA ribosomal RNA gene database project: improved data processing and web-based tools. Nucleic Acids Res. 41, D590–D596. 10.1093/nar/gks1219 23193283 PMC3531112

[B41] R Development Core Team (2013). R: A Language and Environment for Statistical Computing. Vienna: R Foundation for Statistical Computing Available online at: http://www.R-project.org

[B42] Reis V. M. Estrada-de los Santos P. Tenorio-Salgado S. Vogel J. Stoffels M. Guyon S.. (2004). *Burkholderia tropica* sp. nov., a novel nitrogen-fixing, plant-associated bacterium. Int. J. Syst. Evol. Microbiol. 54, 2155–2162. 10.1099/ijs.0.02879-0 15545451

[B43] Rodrigues J. L. Pellizari V. H. Mueller R. Baek K. Jesus Eda C. Paula F. S.. (2013). Conversion of the Amazon rainforest to agriculture results in biotic homogenization of soil bacterial communities. Proc. Natl. Acad. Sci. U.S.A. 110, 988–993. 10.1073/pnas.1220608110 23271810 PMC3549139

[B44] Rousk J. Bååth E. Brookes P. C. Lauber C. L. Lozupone C. Caporaso J. G.. (2010). Soil bacterial and fungal communities across a pH gradient in an arable soil. ISME J. 4, 1340–1351. 10.1038/ismej.2010.58 20445636

[B45] Schneider D. Arp G. Reimer A. Reitner J. Daniel R. (2013). Phylogenetic analysis of a microbialite-forming microbial mat from a hypersaline lake of the Kiritimati atoll, Central Pacific. PLoS ONE 8:e66662. 10.1371/journal.pone.0066662 23762495 PMC3677903

[B46] Shen J. P. Zhang L. M. Di H. J. He J. Z. (2012). A review of ammonia-oxidizing bacteria and archaea in Chinese soils. Front. Microbiol. 3:296. 10.3389/fmicb.2012.00296 22936929 PMC3424668

[B47] Soares-Filho B. S. Nepstad D. C. Curran L. M. Cerqueira G. C. Garcia R. A. Ramos C. A.. (2006). Modelling conservation in the Amazon basin. Nature 440, 520–523. 10.1038/nature04389 16554817

[B48] Spang A. Poehlein A. Offre P. Zumbrägel S. Haider S. Rychlik N.. (2012). The genome of the ammonia-oxidizing Candidatus Nitrososphaera gargensis: insights into metabolic versatility and environmental adaptations. Environ. Microbiol. 14, 3122–3145. 10.1111/j.1462-2920.2012.02893.x 23057602

[B49] Teske A. Sørensen K. B. (2008). Uncultured archaea in deep marine subsurface sediments: have we caught them all? ISME J. 2, 3–18. 10.1038/ismej.2007.90 18180743

[B50] Tischer A. Potthast K. Hamer U. (2014). Land-use and soil depth affect resource and microbial stoichiometry in a tropical mountain rainforest region of southern Ecuador. Oecologia 175, 375–393. 10.1007/s00442-014-2894-x 24532178

[B51] Tripathi B. M. Kim M. Lai-Hoe A. Shukor N. A. Rahim R. A. Go R. (2013). pH dominates variation in tropical soil archaeal diversity and community structure. FEMS Microbiol. Ecol. 86, 303–311. 10.1111/1574-6941.12163 23773164

[B52] Tripathi B. M. Kim M. Singh D. Lee-Cruz L. Lai-Hoe A. Ainuddin A. N. (2012). Tropical soil bacterial communities in Malaysia: pH dominates in the equatorial tropics too. Microb. Ecol. 64, 474–484. 10.1007/s00248-012-0028-8 22395784

[B53] Tripathi B. M. Lee-Cruz L. Kim M. Singh D. Go R. Shukor N. A.. (2014). Spatial scaling effects on soil bacterial communities in Malaysian tropical forests. Microb. Ecol. 68, 247–258. 10.1007/s00248-014-0404-7 24658414

[B54] Wang Y. Qian P. Y. (2009). Conservative fragments in bacterial 16S rRNA genes and primer design for 16S ribosomal DNA amplicons in metagenomic studies. PLoS ONE 4:e7401. 10.1371/journal.pone.0007401 19816594 PMC2754607

[B55] Wemheuer B. Taube R. Akyol P. Wemheuer F. Daniel R. (2013). Microbial diversity and biochemical potential encoded by thermal spring metagenomes derived from the Kamchatka Peninsula. Archaea 2013, 136714. 10.1155/2013/136714 23533327 PMC3600328

[B56] Zhou J. Wu L. Deng Y. Zhi X. Jiang Y. H. Tu Q.. (2011). Reproducibility and quantitation of amplicon sequencing-based detection. ISME J. 5, 1303–1313. 10.1038/ismej.2011.11 21346791 PMC3146266

