# Supplementary material for: Impact of Lowland Rainforest Transformation on Diversity and Composition of Soil Prokaryotic Communities in Sumatra (Indonesia)
Source: Front Microbiol. 2015 Dec 8;6:1339. doi: 10.3389/fmicb.2015.01339 (PMC4672069; doi:10.3389/fmicb.2015.01339)
Supplement: Table S2 — Statistical analyses of soil characteristics. [file Table2.PDF]

Table S2. Statistical analyses of soil characteristics. Shown are mean values of soil properties and standard deviation for each land use type and ANOVA *P* values. Differences of soil properties between land use types were analyzed by employing ANOVA of variance and Tukey's HSD comparisons. Significant ANOVA *P* values are shown in bold (P < 0.05). Means with the same letter are not significantly different. Values with grey background were not normal distributed (Shapiro-Wilk normality test P < 0.05), data were log transformed. Detailed soil and site information for all 32 core sampling sites is provided in Supplementary Table S1.

**Soil property**	**Land use system**								**ANOVA**
	**Rainforest**		**Jungle rubber**		**Rubber**		**Oil palm**		***P***
pH	4.22 ±0.05	b	4.43 ±0.06	a	4.45 ±0.15	a	4.45 ±0.07	a	**0.001**
P (mg/kg soil)	9.3 ±4.93	ab	7.4 ±2.49	ab	4.48 ±1.5	b	16.68 ±10.67	a	**0.016**
N (mg/kg soil)	2253.76 ±711.12		2634.56 ±847.75		1816.8 ±387.99		2149.97 ±706.03		0.337
C (g/kg soil)	29.81 ±6.25		36.35 ±11.34		23.88 ±6.14		27.85 ±9.41		0.164
basal respiration (µg O_2*h^{-1}*g soil dw^{-1})	6.28 ±1.17		6.11 ±1.66		4.11 ±0.80		4.51 ±1.49		**0.027**
microbial biomass (µg Cmic*g soil dw^{-1})	476.3 ±94.19		572.56 ±155.56		434.46 ±104.09		441.72 ±116.23		0.286
H_2O (% of dry weight)	75.45 ±44.46		86.95 ±46.96		71.46 ±32.33		62.41 ±34.79		0.776
Silt (%)	28.6 ±5.90		26.61 ±9.67		26.74 ±8.04		23.28 ±7.01		0.673
Clay (%)	31.77 ±7.57	b	45.1 ±12.60	ab	40.94 ±10.22	ab	56 ±15.00	a	**0.017**
