# Supplementary material for: Impact of Lowland Rainforest Transformation on Diversity and Composition of Soil Prokaryotic Communities in Sumatra (Indonesia)
Source: Front Microbiol. 2015 Dec 8;6:1339. doi: 10.3389/fmicb.2015.01339 (PMC4672069; doi:10.3389/fmicb.2015.01339)
Supplement: Figure S1 — Box plots of soil characteristics summarized by land use system. [file Image1.PDF]

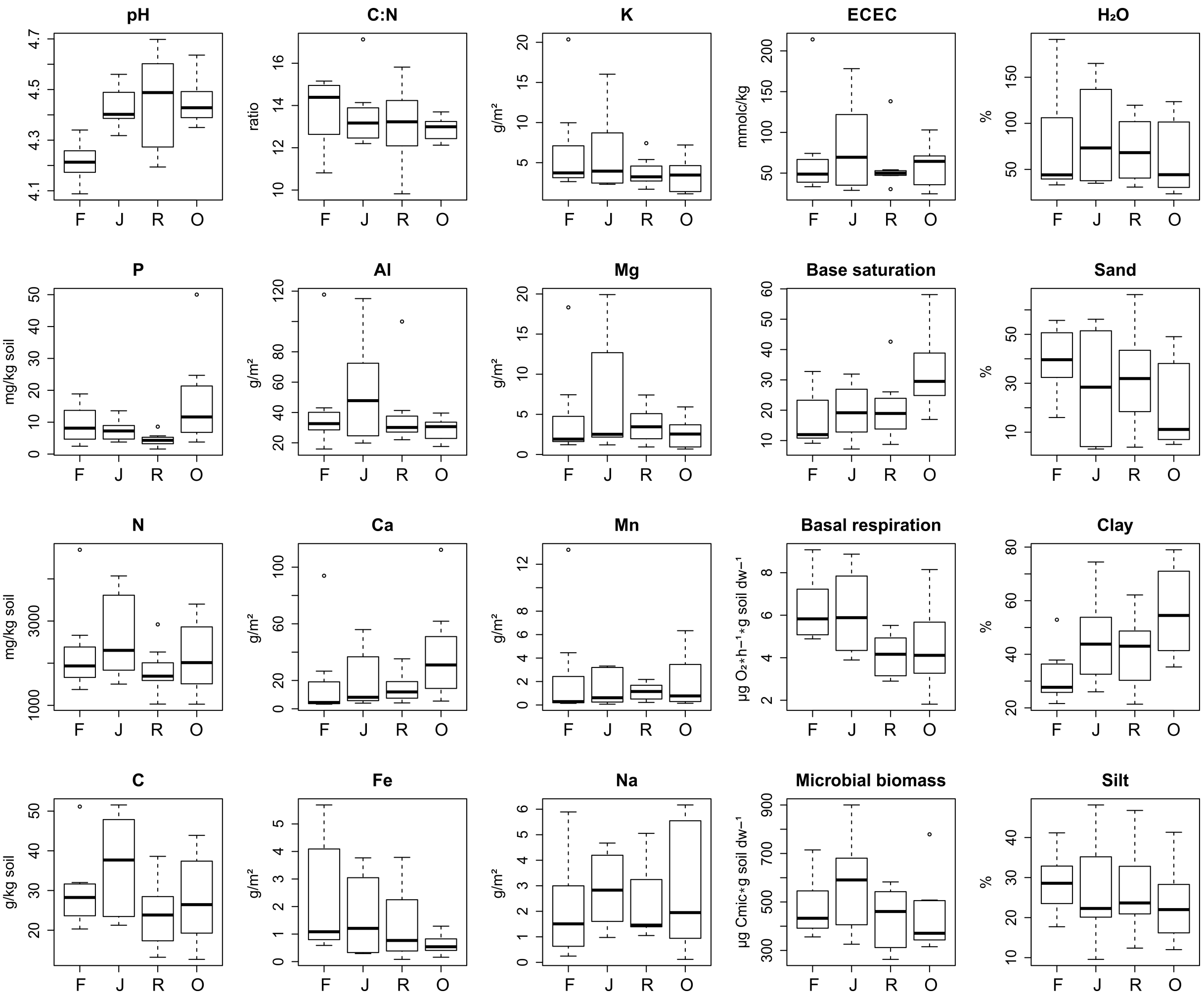

FIGURE S1. Box plots of soil characteristics analyzed summarized by land use system (for details on core plot level, see Supplementary Table S1). F, rainforest; J, jungle rubber; R, rubber plantation; O, oil palm plantation; ECEC, effective cation exchange capacity.
