# Supplementary material for: Impact of Lowland Rainforest Transformation on Diversity and Composition of Soil Prokaryotic Communities in Sumatra (Indonesia)
Source: Front Microbiol. 2015 Dec 8;6:1339. doi: 10.3389/fmicb.2015.01339 (PMC4672069; doi:10.3389/fmicb.2015.01339)
Supplement: Figure S3 — Rarefaction analyses of the bacterial diversity of the two analyzed landscapes and four land use systems. [file Image3.PDF]

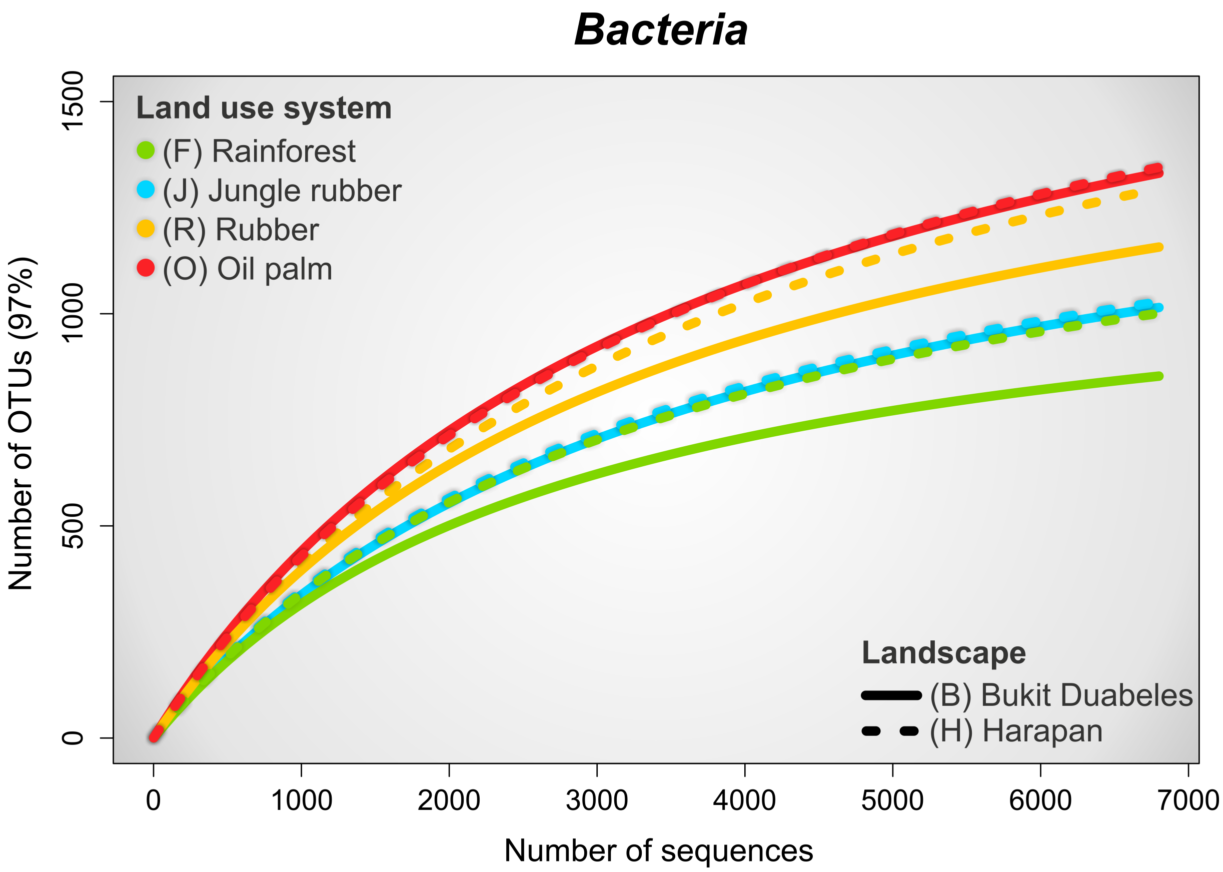

FIGURE S3. Rarefaction analyses of the bacterial diversity of the two analyzed landscapes and four land use systems. Clustering was performed at 97% genetic identity. The results of all analyzed samples of each land use system in a landscape were summarized and averaged (for diversity metrics at subplot level, see Supplementary Table S3).
